# Investigating the role of task relevance during rhythmic sampling of spatial locations

**DOI:** 10.1038/s41598-023-38968-z

**Published:** 2023-08-05

**Authors:** Olof J. van der Werf, Teresa Schuhmann, Tom de Graaf, Sanne Ten Oever, Alexander T. Sack

**Affiliations:** 1 Section Brain Stimulation and Cognition, Department of Cognitive Neuroscience, Faculty of Psychology and Neuroscience, Maastricht University, Oxfordlaan 55, 6229 EV Maastricht, The Netherlands; 2 Maastricht Brain Imaging Centre (MBIC), Maastricht University, Maastricht, The Netherlands; 3 Language and Computation in Neural Systems Group, Max Planck Institute for Psycholinguistics, Nijmegen, The Netherlands; 4 Donders Centre for Cognitive Neuroimaging, Radboud University, Nijmegen, The Netherlands; 5 Department of Psychiatry and Neuropsychology, School for Mental Health and Neuroscience (MHeNs), Brain and Nerve Centre, Maastricht University Medical Centre+ (MUMC+), Maastricht, The Netherlands

**Keywords:** Cognitive neuroscience, Sensory processing

## Abstract

Recently it has been discovered that visuospatial attention operates rhythmically, rather than being stably employed over time. A low-frequency 7–8 Hz rhythmic mechanism coordinates periodic windows to sample relevant locations and to shift towards other, less relevant locations in a visual scene. Rhythmic sampling theories would predict that when two locations are relevant 8 Hz sampling mechanisms split into two, effectively resulting in a 4 Hz sampling frequency at each location. Therefore, it is expected that rhythmic sampling is influenced by the relative importance of locations for the task at hand. To test this, we employed an orienting task with an arrow cue, where participants were asked to respond to a target presented in one visual field. The cue-to-target interval was systematically varied, allowing us to assess whether performance follows a rhythmic pattern across cue-to-target delays. We manipulated a location’s task relevance by altering the validity of the cue, thereby predicting the correct location in 60%, 80% or 100% of trials. Results revealed significant 4 Hz performance fluctuations at cued right visual field targets with low cue validity (60%), suggesting regular sampling of both locations. With high cue validity (80%), we observed a peak at 8 Hz towards non-cued targets, although not significant. These results were in line with our hypothesis suggesting a goal-directed balancing of attentional sampling (cued location) and shifting (non-cued location) depending on the relevance of locations in a visual scene. However, considering the hemifield specificity of the effect together with the absence of expected effects for cued trials in the high valid conditions we further discuss the interpretation of the data.

## Introduction

We are constantly bombarded with an overwhelming amount of visual information and stimuli competing for our attention. The brain cannot process all this visual information simultaneously due to limitations in processing and representational capacities. Fortunately, not all visual information is equally relevant for our behavioural goals. Visuospatial attention is the processing filter by which our brain prioritizes processing of visual stimuli in certain locations in space over others, for instance because those locations are behaviourally relevant^1^. Neurocognitive processes underlying visuospatial attention bias the processing of visual stimuli based on both salience (‘capturing’ attention) and behavioural relevance (prompting voluntary attention allocation)^2^.

In recent years it has become clear that visuospatial attention is not stably employed towards one location or visual field over time, but rather samples visual information rhythmically at theta (4–8 Hz) to alpha (8–12 Hz) frequencies^3–8^. In behavioural paradigms, rhythmic attentional sampling results in a periodic, waxing-and-waning pattern of detection performance of stimuli at a particular location^5^. Neurophysiological evidence for rhythmic attention is converging from electrophysiological measurements such as magneto- or encephalography (M/EEG)^9,10^ and interventional techniques such as transcranial magnetic stimulation (TMS)^11^. It was shown that rhythmic patterns of visual detection tightly map onto neuronal oscillations in visual and attentional areas^3,12–14^ that seem to originate in subcortical areas^15^.

VanRullen^6,8^ proposed that behaviourally observable rhythms in visuospatial attention emerge from a fundamental 8-Hz information sampling mechanism. This mechanism coordinates periodic windows for, on the one hand, *exploitation* (i.e. sensory sampling of a spatial location) and, on the other hand, *exploration* (i.e. shifting towards other locations)^4^. Interestingly, the rhythmic pattern of exploration seems to be directly proportional to the number of locations^6,16,17^. In a visual scene involving two potential target locations, one in each visual field, this results in a waxing-and-waning perceptual sensitivity at a single location at a 4-Hz rhythm^5,11,18–20^. In the case of three relevant target locations, each location is sampled at ~ 2.7 Hz^16^. This implies that the rhythmic attentional spotlight shifts from location to location, sampling each location across the fundamental rhythm of 8 Hz.

This principle holds when each location in the visual scene is equally relevant, but often, that is not the case. What if behavioural goals favour attending one location over other locations?

One important objective for visuospatial attention is to match the allocation of attention (‘weight’) across spatial locations to their task-dependent behavioural relevance^21,22^. It is thought that the relationship between sampling and shifting changes based on the allocation of behavioural relevance of spatial locations in a visual scene^4,12^. Consider a visual scene involving two spatial locations: when these spatial locations bear equal behavioural relevance, frequent exploratory attentional shifts between the two locations are to be expected. Conversely, when behavioural goals favour one location, only *occasional* attentional shifts towards the other location are expected^4^. It is thought that exploration of spatial locations is the default mode of the visuospatial attentional system, whereas exploitation of one location is thought to require top-down attentional influence^12^.

We previously tested the effect of cue validity on rhythmic sampling of spatial locations, using a modified Egly-Driver task^23^. The Egly-Driver task is designed to test object- and space-based attention and consists of a exogenous spatial cue, highlighting the to-be-attended location with a black outline around the end of one of two bars, which are oriented vertically or horizontally^24^. The observer is instructed to respond to a slight change in luminance, located on the cued or a non-cued location either on the other end of the same bar or on the equidistantly located end of the other bar^24^. We modified the time between the cue and the target to investigate the (sinusoidal) relationship between cue-target interval and accuracy of target detection; we also modified the predictability of the cue (33%, 80% (as in^20^) or 100%). We did not find an effect of behavioural relevance of spatial locations on rhythmic attention^23^. However, the task recruits both bottom-up and top-down visuospatial attention. Task relevance (manipulated by cue validity) recruits top-down attention to a greater extent than bottom-up attention^25^. Therefore, the modified Egly-Driver task might not have provided the most ideal circumstances to assess the effect of cue validity on rhythmic attention. A task involving a purely top-down attentional component might be a more suitable option to test for the effects of rhythmic attention.

**Figure 1:**
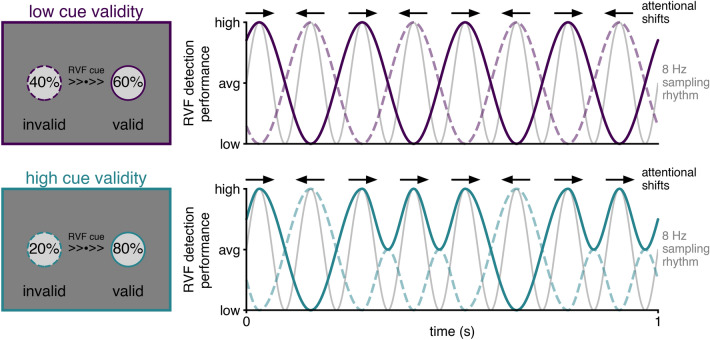
Hypothesis figure. Schematic representation of hypothesized performance over time in conditions where the cue validity is low (top) or high (bottom) illustrated for the right visual field (RVF). The solid lines represent detection performance for validly cued trials, the dashed lines represent performance for invalidly cued trials (‘avg’ = average). The grey line represents the underlying 8 Hz sampling rhythm or ‘clock’, providing periods of heightened or diminished perceptual sensitivity. In periods of heightened sensitivity, attentional shifts (represented by the black arrows) could occur towards either visual field. When the cue validity is low, there is a more balanced allocation of attentional resources, leading to as many attentional shifts towards the validly cued location in the RVF as the invalidly cued location in the LVF (left visual field). This results in a ~ 4 Hz rhythm of performance on the task over time. When the cue validity is high, however, the task relevance of the validly cued location is much higher than the task relevance of the invalid location. As a result, there are attentional shifts towards the validly cued RVF in almost every moment of heightened sensitivity. These shifts towards the validly cued location render reorienting from the valid towards the invalid location more challenging, resulting in a ~ 8 Hz detection performance over time for valid and invalid trials.

Here, we investigated whether rhythmic attentional orienting towards a spatial location depends on a top-down monitoring of the behavioural relevance of the sampled locations in the visual scene. To test this empirically, we employed an orienting task with an endogenous cue, in which participants were asked to respond to grating stimuli presented in the left and right visual fields. We made use of an arrow cue, with the goal to recruit top-down attentional deployment towards one location. Crucially, the cue-to-target interval length was systematically varied, resulting in a ‘time course’ of behavioural performance, which represents the reaction times for each interval length. We manipulated behavioural relevance of each of the two locations in this task by altering the cue validity. The cue validity is the ratio between valid cues (correctly predicting the target location) and invalid cues (incorrectly predicting the target location). A high cue validity prompts a higher attentional engagement towards the cued location. Namely, because it results in faster responses to cued targets and slower responses to non-cued targets, i.e. a higher *reorienting cost*^26,27^. Here, we compared three cue validities: 60%, 80% and 100%. We hypothesise that the behavioural relevance of a spatial location dictates the pattern of endogenous rhythmic attentional orienting towards that location (Fig. 1). Namely, a lower behavioural relevance of the cued location would result in a very frequent attentional shifts towards the non-cued location. In this case, the fundamental 8 Hz sampling mechanism would be close-to-equally divided, because there is little difference in behavioural relevance between the two locations. We expect that both the cued and the non-cued location will then be sampled at 4 Hz. Conversely, a higher behavioural relevance would result in more stable attentional sampling at only the cued location. This would manifest as an 8 Hz rhythm of performance at the cued location. In our a-priori analysis plan, we focus on the analysis on the cued location to ensure sufficient trial amounts (288 valid trials per condition per participant, 9216 trials in total) and only look at non-cued targets in a post-hoc exploratory analysis.

## Methods

### Participants

A total of 32 participants (mean age: 23; range: 19–28, 22 females) participated in the study. Previous studies resulting in significant behavioral oscillatory effects used less participants and a similar number of trials^28,29^, suggesting that we have sufficient power. All participants were right-handed and had normal or corrected-to-normal vision. Six participants were excluded due to a high number of eye blinks and / or saccades (above 20%). The study was approved by the Ethics Review Committee Psychology and Neuroscience (ERCPN) at Maastricht University, The Netherlands (ethical approval number: OZL- 177_03_03_2017_S12) and in concordance with the World Medical Association Declaration of Helsinki. All participants gave their written informed consent before participating in the study. Participants were compensated for their time with a monetary reward or participation credits.

### Procedure

Participants were seated in front of a gamma-corrected PC monitor (Iiyama ProLite, aspect ratio of 1920 × 1080 and a refresh rate of 60 Hz), in a lightly dimmed room. Viewing distance was kept stable at 57 cm from the monitor by means of a chin rest. We performed video-based monocular eye tracking at 1000 Hz with the Eyelink1000 system (SR Research, Mississauga, Ontario, Canada). A standard nine-point calibration and validation procedure was used to calibrate the eye tracker. After calibration of the eye tracker, participants were familiarized with the task using a practice block (60 trials, at a cue validity of 80%). Participants were asked to keep their head in the chin rest and to blink only after giving their response after each trial.

### Design

**Figure 2:**
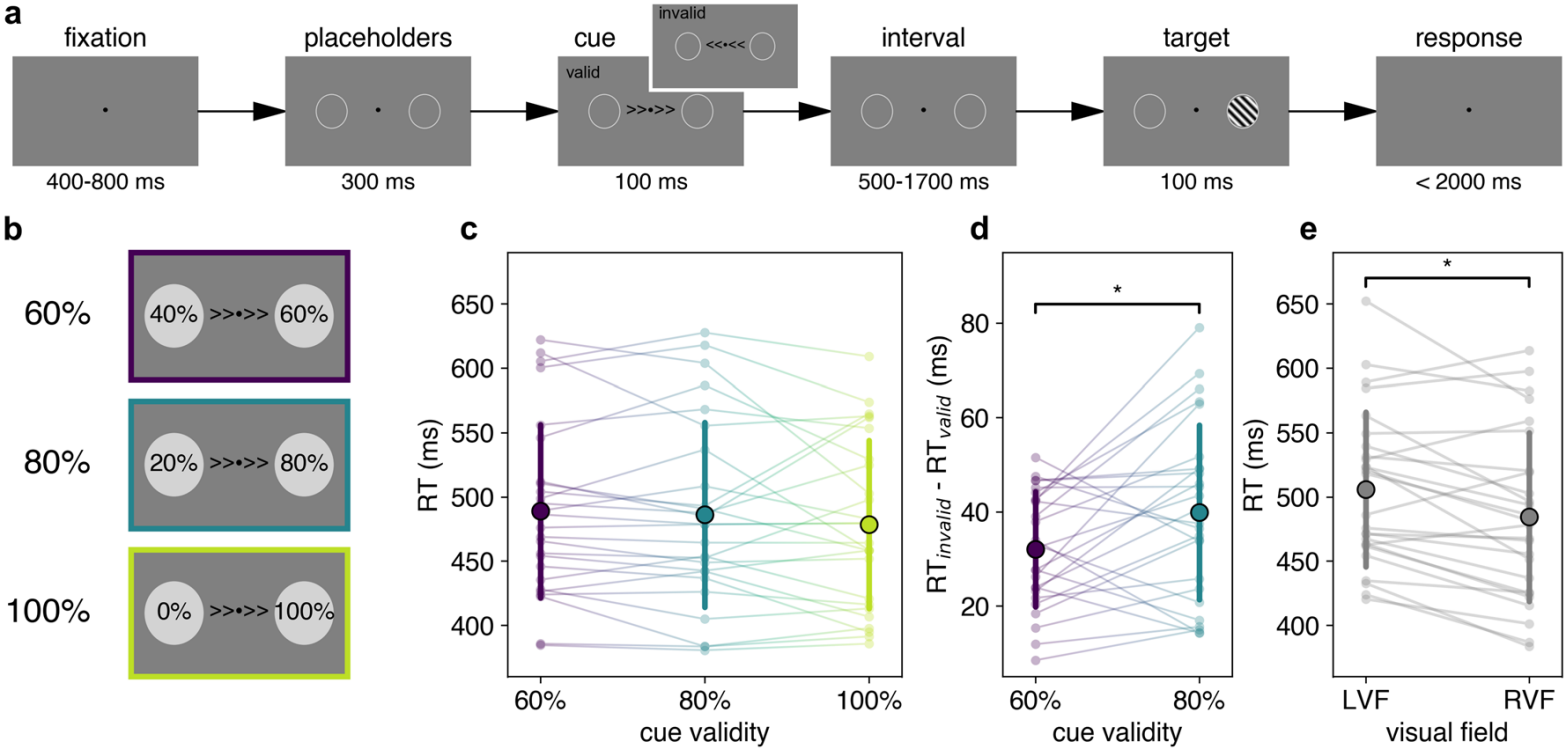
Overview of methods. (**a**) Schematic overview of a single trial. Trials started with the appearance of a central fixation dot; participants were asked to fixate on this dot throughout a trial. After this initial fixation period of 400–800 ms (jittered duration), two placeholders appeared on each side of the fixation cross. After 300 ms, a central cue appeared for a duration of 100 ms. This cue could be valid (correctly predicting the upcoming stimulus location) or invalid. Then, after a variable cue-target interval (500–1700 ms), a Gabor patch appeared in one of the visual fields for 100 ms. Participants were asked to indicate the orientation (45° or – 45°) of the grating stimulus as fast and accurately as possible, within a response window of 1500 ms. (**b**) Schematic overview of cue validity conditions and likelihood of target appearance (in %) in each location. Note that these numbers are reversed when the cue points towards the left. (**c**–**d**) Overall behavioural results in each condition confirm that behavioural relevance of locations was successfully controlled using cue validity. (**c**) Reaction times on valid trials for each cue validity condition. Reaction times did not differ significantly between cue validity conditions, as shown by a Repeated Measures ANOVA (*P* = 0.063). (**d**) The cost of reorienting (RT_valid_ – RT_invalid_) was significantly higher in the high (80%) cue validity condition than in the low (60%) cue validity. Single asterisks (*) denote significance where *P* < 0.05. (**e**) Reaction times to validly cued targets, split per hemifield.

Participants performed a version of an endogenous orienting task^30,31^, which was modified to investigate participant responses across different cue-target interval bins (Fig. 2). Stimuli were presented on a grey background (luminance: 71.8 cd/m^2^). Participants were asked to maintain fixation on a central dot (•, diameter 0.15° visual angle) throughout the task. Each trial started with a fixation period, with a jittered duration between 400 and 800 ms (ms), after which two white circular placeholders (luminance: 144.0 cd/m^2^, line width: 2 pixels, size 5°, eccentricity 7° visual angle from centre) appeared. After 300 ms, a centrally located cue appeared for a duration of 100 ms. This endogenous cue pointed either towards the left (< < • < <) or towards the right (> > • > >). There were two cue types: valid cues predicted the upcoming target location correctly, whereas invalid cues pointed towards the opposite visual field as the upcoming target. The probability that the cue correctly predicts the upcoming target location, referred to as *cue validity*, was 60%, 80% or 100%. The cue-to-target interval duration was varied between 500 and 1683.33 ms, in 72 steps of 16.67 ms. The target was located either in the left or in the right visual field and oriented 45° or − 45°. It consisted of a Gabor patch (diameter: 5°, eccentricity: 7° visual angle from centre, SD: 0.75, Michelson contrast: 0.60, spatial frequency: 1.5 cycles / degree). Participants were asked to report the orientation of the Gabor patch as fast and accurately as possible, by pressing a button on a custom-made button box with the right hand (left button for 45°, right button for − 45°). The maximum time to give a response was 1500 ms. Task stimuli were programmed using the Psychophysics Toolbox (PsychToolbox)^32^ in MATLAB (MathWorks, version 2018b). The task was divided into six blocks, two blocks per cue validity condition (60%, 80%, 100%). Before each block and in every small break (after 50 trials), participants were informed about the current cue validity percentage, to encourage them to adopt an appropriate attentional strategy. At shorter cue-target intervals, the likelihood that that any cue, even a symbolic cue, elicits merely an exogenous orienting response, is high^33^; however for delays higher than 300 ms, this effect vanishes. Therefore, the cue-target delays in this study are set to be longer (500–1700 ms) to increase the likelihood that the task is recruiting endogenous attention. Trials were pseudo-randomly allocated so that each of the 72 temporal bins contained 2 validly cued trials per visual field (thus, 4 in total); the amount of invalidly cued trials per bin varied depending on cue validity. In total, each cue validity condition consisted of 288 validly cued trials, and therefore a different total number of trials per condition (480, 360 and 288 for the 60%, 80% and 100% cue validity, respectively). Temporal bins were randomly distributed across the experimental blocks within each cue validity condition.

### Data analysis

Data analysis and visualisation was conducted in Python (version 3.8) and documented using Jupyter Notebooks. The single-trial least squares spectrum (stLSS) analysis is conducted using code from Tosato et al.^34^, translated from MATLAB into Python. Jupyter Notebooks containing code, explanations of each analysis step and additional plots can be found at https://github.com/olofvanderwerf/rhythmic-attention. Furthermore, the “readme” file contains links to interactive executable Notebooks in Binder, allowing for easy access and reproduction of analysis steps (for a discussion on this topic see^35^).

#### Pre-processing

**Figure 3:**
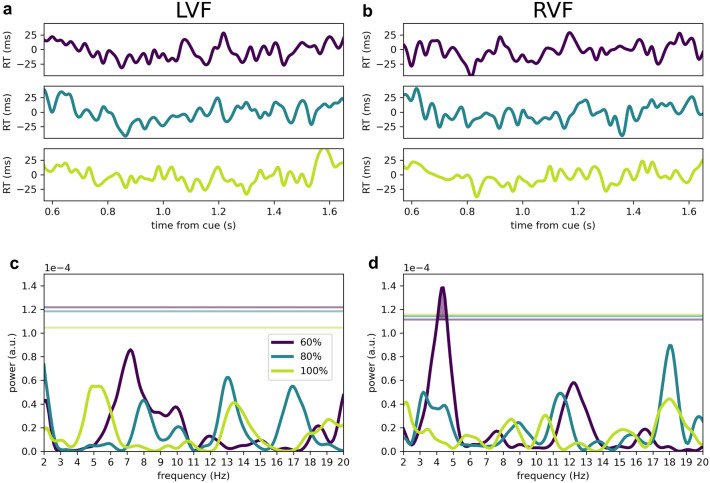
Attentional orienting is rhythmic, depending on cue validity (**a**–**b**) Time courses of reaction times across cue-to-target intervals (mean ± standard error of measurement (SEM)) at validly cued locations on the LVF (**a**) and the RVF (**b**). Time courses are constructed using a Gaussian moving kernel across cue-to-target intervals. (**c**–**d**) Power spectra respectively corresponding to the time courses in A and B, as yielded from the stLSS analysis. Horizontal lines in corresponding colours denote the 95^th^ percentile of the maximum permutation spectrum (i.e. Max-Based correction for multiple comparisons). Reaction times to targets in the RVF fluctuated significantly at ~ 4 Hz in the low cue validity (60%) condition (*P*_peak_ = 0.013 at 4.4 Hz).

Validly cued trials with blinks (absence of a tracked eye for more than 100 ms) and saccades (exceeding 2° of visual angle), and trials with incorrect responses were excluded from the analysis (84 trials (7%**)** on average). Because of the differences between visual fields in terms of overall behaviour, and based on previous literature^5,36^ we decided to consider temporal fluctuations of reaction times (RT) for each visual field separately. For each visual field (left, right or both) and condition (60%, 80% or 100%), trials were pooled across participants (i.e. the ‘aggregate observer’). We convoluted these trials in the time domain using a Gaussian kernel (sigma = 0.01 s), resulting in an artificial time course of RT across cue-to-target interval (Fig. 3a,b). We obtained the linear trend from this artificial time course and subtracted the trend from each single trial’s RT. Lastly, we applied a Hanning taper by multiplying each trial’s RT with the value of the taper at the corresponding cue-to-target interval.

#### Analysis

For each visual field and condition, we applied the Single-Trial Least Square Spectrum (stLSS) analysis method, the most sensitive and specific method for such data as proposed by Tosato et al.^34^ (see also^29,37^ for implementations of this method). This method calculates a Generalised Linear Model (GLM) per frequency of interest, predicting, for each trial, the RT (dependent variable) given the trial’s cue-to-target interval (independent variable). A sine and a cosine are used as predictors, per frequency between 2 and 20 Hz in steps of 0.1 Hz. The regression coefficients belonging to the sine and cosine are used to approximate the Fourier coefficient, of which the absolute values are squared to obtain the power spectrum. Next to the stLSS analysis, we used a more conventional approach in which we convoluted trials on the participant-level using a boxcar (width 50 ms, in steps of ~ 16.67 ms). We then applied a Fast Fourier Transform (FFT) on the group-averaged time course and assessed spectral peaks by comparing it to a surrogate power spectrum (1000 permutations, FDR-corrected). This analysis provided similar results (see Supplementary Methods).

#### Statistics

To statistically assess our overall behavioural results, we ran a two-way Repeated Measures Analysis of Variance (RM-ANOVA) with factors *cue validity* (60%, 80%, 100%) and *visual field* (LVF, RVF) on mean reaction times (RT) in the validly cues trials. Results were Greenhouse-Geiser corrected. We ran follow-up pairwise comparisons and Bonferroni-corrected the outcomes. We also ran a paired t-test to assess the difference in *reorienting cost* (RT_valid_–RT_invalid_) between 60 and 80% cue validity (the 100% cue validity condition is not included because it contains no invalidly cued trials). To investigate statistical significance of the found spectral peaks, we applied non-parametric permutation testing. First, we tested whether, for each cue validity condition, there was a significant fluctuation of performance over time. Here, the null hypothesis states that there is no temporal pattern in performance, thus we shuffled the temporal bin labels. Second, we tested whether rhythmic attention is different across cue validity conditions. Here, the null hypothesis states that there is no difference in cue validity, thus we shuffled the condition labels of the valid trials, for each visual field separately. We repeated this 1000 times, so that for each visual field and condition there was a surrogate distribution of shuffled temporal bin labels and condition labels. We applied the same analysis steps (see above) and ended up with a surrogate distribution of 1000 power spectra, to which we could compare the observed power spectrum. We used the Max-Based correction for multiple comparisons^34,38^ which works as follows: we determined the maximum power for each permutation to obtain a distribution of maximum values. Next, we compared the observed power spectrum to this distribution to obtain the p-value for each frequency.

We applied two further analyses on our results. To shed light on the influence or bias of each individual participant on our results, we repeated the analysis for our significant result(s) while leaving individual participants out. We compared the resulting power spectra for each ‘one-out’ analysis to its respective permutation spectrum. In this case, this resulted in 26 outcomes: a range of p-values per frequency for each ‘one-out’ analysis. Besides this, we applied an analysis posed by Brookshire^39^ on our data, to decipher the effects of autocorrelation in behavioural oscillations, which gets destroyed by shuffling the trials in the time domain.

## Results

### Effects of cue validity on attentional orienting

First, we investigated whether we successfully altered the task relevance of target location by altering the cue validity. To this aim, we conducted a two-way RM ANOVA to assess the effect of within-participant factors *cue validity* (60%, 80%, 100%) and *visual field* (LVF, RVF) on mean reaction times (RT; collapsed across cue-target interval bins). Note that we could not add *cue type* (valid vs. invalid) as a factor to this analysis because of a lack of invalid trials in the 100% cue validity condition (we only report on valid trials here). There was no statistically significant effect of *cue validity* on reaction times after Greenhouse-Geiser correction (*F*_2,50_ = 3.25, *P* = 0.063, η^2^ = 0.11, Fig. 2c). There was a statistically significant effect of *visual field* on reaction times (*F*_1,25_ = 13.18, *P*_corr_ = 0.001, η^2^ = 0.35) as well as a statistically significant interaction effect between *visual field* and *cue validity* (*F*_2, 50_ = 3.31, *P*_corr_ = 0.0488, η^2^ = 0.12). Mean reaction times towards validly cued targets in the RVF (484 ± 64 ms) were significantly faster than reaction time to targets in the LVF (506 ± 59 ms, *T* = 3.68, *P* = 0.001, *d* = 0.34, Fig. 2e). One-way RM ANOVAs for each separate visual field found a statistically significant difference in RT across cue validity for the LVF (*F*_2.0, 50.0_ = 5.32, *P* = 0.008, η^2^ = 0.18) but not for the RVF (*F*_2.0, 50.0_ = 1.32, *P* = 0.28, η^2^ = 0.05; Supplementary Figure S1). Bonferroni-corrected follow-up pairwise comparisons for the LVF revealed a statistically significant difference between the 60% (516 ± 66 ms) and 100% conditions (495 ± 57 ms; *T* = 3.03, *P* = 0.005, *d* = 0.34), but not between 60 and 80% (506 ± 64 ms; *T* = 1.79, *P* = 0.08, *d* = 0.14) nor between 80 and 100% (495 ± 57 ms, *T* = 1.66, *P* = 0.11, *d* = 0.19). In terms of accuracy, no significant effect was found for *cue validity* in a two-way RM ANOVA (*F*_2, 50_ = 0.63, *P*_corr_ = 0.53, η^2^ = 0.03), nor for *visual field* (F_1, 25_ = 0.75, *P*_corr_ = 0.40, η^2^ = 0.03), nor for the interaction effect (*F*_2, 50_ = 0.70, *P*_corr_ = 0.48, η^2^ = 0.03). Our following analysis focused on the attentional cost of reorienting, which could only be calculated for the 60% and 80% conditions as it requires invalid cued trials (calculated by RT_invalid_–RT_valid_). Using a paired-samples t-test, we found that the reorienting cost from the cued towards the non-cued location was significantly higher at 80% cue validity (40 ± 18 ms) than at 60% cue validity (32 ± 12 ms, *T* = − 2.51, *P* = 0.019, *d* = 0.50, Fig. 2d). If our manipulation was successful in altering participants’ attentional allocation based on cue validity, we would have expected a significant effect for the valid trials and reorienting costs for both hemi fields. While we do find and effect of reorienting across hemi fields, the valid trial analysis only showed an effect for the LVF. The main effect of cue validity for the valid trials only showed a trend, which does not fully match our manipulation checks. Nonetheless, we went to proceed with our pre-planned analysis strategy.

### Main rhythmic sampling analysis

We analysed temporal fluctuations in reaction times on validly cued trials using non-parametric permutation testing, where we shuffled temporal bin labels. The spectral profile of RVF attentional performance over time revealed a significant spectral peak at ~ 4 Hz (range: 4.1–4.6 Hz, *P*_peak_ = 0.016, Max-Based corrected, at 4.4 Hz) for the 60% cue validity condition, but no significant peaks for the 80% or 100% cue validity conditions (all *P* > 0.10, Fig. 3d). Periodicities in attentional performance on valid trials in the LVF showed a peak at 7.2 Hz in the 60% cue validity condition, albeit not significant after Max-Based correction (*P*_peak_ = 0.23, Max-Based corrected, Fig. 3c). As with the RVF, no significant peaks were found for the 80% and 100% cue validity conditions in the LVF (all *P* > 0.10).

We tested more directly whether the significant 4 Hz effect dependent on cue validity. To this aim, we employed non-parametric permutation testing where we shuffled the cue validity labels (60%, 80%, 100%) across trials. In the RVF, for the 60% cue validity condition, the peak at 4.4 Hz in the power spectrum was also significant compared to the condition permutation distribution (4.1–4.5 Hz, *P*_peak_ = 0.015), further corroborating the specificity of our findings to low validity at RVF only. We did not find any statistically significant peaks in the other cue validities in the RVF (all *P* > 0.10), nor any peaks in the LVF (all *P* > 0.10).

We, furthermore, conducted two confirmatory analyses. To investigate the potentially biasing effects of individual subjects, we repeated the analysis leaving each participant out. The results suggest that no individual participant drastically altered the results (range of *p*: *p* = 0.003 to *p* = 0.088; *p*_mean_ = 0.024; range of peak frequencies: 4.3 Hz–4.4 Hz; *p* where *p* > 0.05: *p* = 0.088, *p* = 0.067). The analysis proposed by Brookshire (2022), i.e. fitting an autoregressive (AR(1)) model to the time series, resulted in a a peak at 5.0 Hz, although it did not survive correction for multiple comparisons by False Discovery Rate (FDR; p_uncorr_ = 0.04, p_corr_ = 0.38).

### Rhythmic sampling analysis on invalid trials

**Figure 4:**
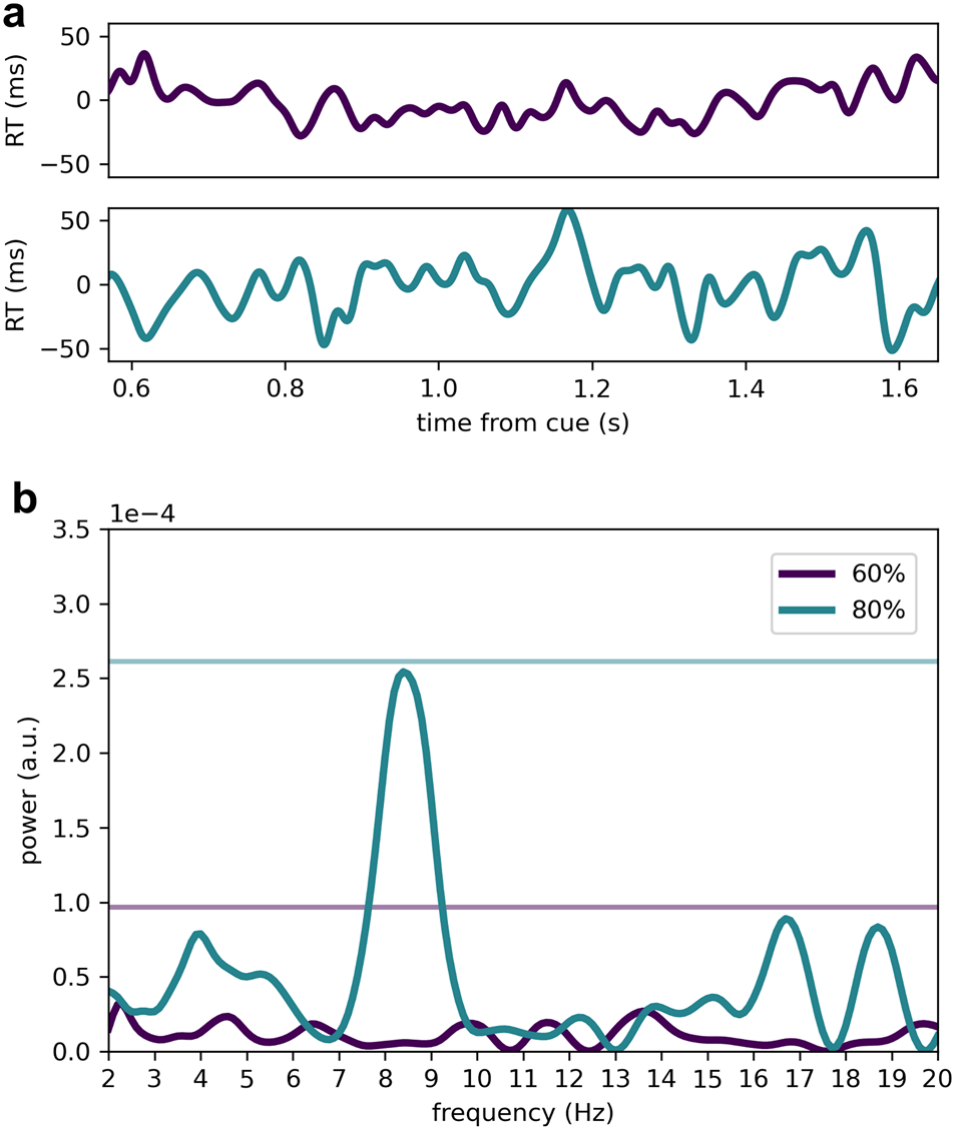
Rhythmic properties of attentional reorienting. (**a**) Constructed time courses of reaction times across cue-to-target intervals in invalid trials, collapsed across visual fields (mean ± SEM), where purple represents 60% cue validity and blue represents 80% cue validity. (**b**) Power spectra respectively corresponding to the time course in A, as yielded by the stLSS analysis. Horizontal lines in corresponding colours denote the 95^th^ percentile of the maximum permutation spectrum (i.e. Max-Based correction for multiple comparisons). There is nearly significant ~ 8 Hz peak in the power spectrum of the 80% condition on the RVF (*P*_peak_ = 0.057 at 8.4 Hz, Max-Based corrected for multiple comparisons).

While our hypothesis regarded the validly cued trials, we nonetheless performed an analysis on the invalidly cued trials (i.e. attentional reorienting). However, please note that these are post-hoc analysis and hampered by low trial amounts. Therefore, we considered all invalidly cued trials pooled across visual fields (Fig. 4a), because of the scarcity of invalid trials in the 80% cue validity condition (~ 30 trials in each visual field per participant). The spectral profile of reorienting in the 80% cue validity condition showed a strong spectral peak at ~ 8 Hz (*P*_peak_ = 0.057 at 8.4 Hz, Fig. 4b), which was not significant. No spectral peaks are visible in the power spectrum of invalid trials in the 60% cue validity condition (all *P* > 0.10, Fig. 4b). Also splitting by visual field did not reveal significant peaks (all *P* > 0.05, see supplementary Fig. 2).

## Discussion

Visuospatial attention seems to sample locations rhythmically, resulting in a fluctuating detection performance at each location in a theta rhythm around 8 Hz^4–6^. If there are multiple locations in a visual scene, attentional needs to be divided across locations, potentially leading to a rhythmic sampling across relevant locations^5,16^. Indeed, in some tasks, the frequency at which attention rhythmically samples each location, has been found to be proportional to the number of relevant locations. When multiple locations bear equal behavioural relevance, each location should be evenly explored by the attentional sampling mechanism. Conversely, if there is one location with relatively high behavioural relevance, this location should be frequently sampled, whereas sporadic exploratory attentional shifts will occur towards other (less relevant) locations. This effect has been theoretically discussed before^4,12^. We empirically studied this hypothesis before, where we manipulated behavioural relevance by systematically varying cue validity, but we did not find clear effects^23^. This could be due to the task we used, which recruits both object- and space-based attention and is largely associated with bottom-up attention. Here, we used a predominantly top-down spatial attention task to investigate how the behavioural relevance of spatial locations influences rhythmic attentional orienting towards each location. We specifically predicted that, on the one hand, low behavioural relevance of the cue results in a more balanced sampling of the cued and non-cued locations, in turn leading to performance fluctuating at 4 Hz at the cued location (evenly distribution of 8 Hz sampling rhythm). On the other hand, high behavioural relevance leads to more frequent sampling of the cued location and to less frequent shifting towards the non-cued locations. This would give rise to a performance fluctuation at 7–8 Hz at the cued location. Full behavioural relevance (100% cue validity) is expected to lead to full exploitation of the cued location, as shifts to non-cued locations are not necessary.

In our study, we manipulated the behavioural relevance of locations in an endogenous orienting paradigm, by changing the validity of the cue (predicting the target location correctly in 60%, 80% or 100% of the trials). While there were indications that this manipulation changed the attentional allocation of the participants as indicated by a significantly higher reorienting cost in the high cue validity condition (80%) compared the low cue validity condition (60%; also see^26,27^), overall reaction times for the valid trials did not show a main effect of cue validity but rather only showed this effect in the LVF. Therefore, our data does not allow us to solidly verify that participants’ attentional resources were fully modulated as expected from our cue validity manipulation. Nonetheless, we here discuss our results based on our pre-planned rhythmic sampling analyses.

To examine rhythmicity in attentional performance, we systematically varied the length of the cue-to-target interval. We analysed rhythmicity of performance across the cue-to-target interval using stLSS analysis. The stLSS analysis, in combination with a Max-Based correction for multiple comparisons, offers a sensitive and specific analysis for this type of behavioural data^34^. We showed that endogenous attentional orienting operates on a theta-rhythmic pattern, but that its manifestation at the cued location depends on the validity of the cue. Behavioural performance at a validly cued location in the right visual field (RVF) significantly fluctuated at ~ 4 Hz, but only when cue validity was low (60%) and not when it was high (80% or 100%). Overall, no effects were found in the LVF, except for a slight, non-significant peak at ~ 7 Hz in the low cue validity. An exploratory analysis suggests an overall 8-Hz sampling at non-cued locations, regardless of visual field. These results are in line with our original hypothesis (Fig. 1), however, it is unclear why we only find an effect for the RVF and not for the LVF. Another inconsistency is that we would have expected 8 Hz effects for both the valid as well as the invalid trials. We here below discuss and speculate about these inconsistencies.

The 4-Hz peak in the 60% cue validity condition on the RVF suggests that a low behavioural relevance prompts frequent back-and-forth sampling, such that the cued location is sampled only on every second cycle on the 8-Hz sampling mechanism. However, one would then also expect that the non-cued location (after an invalid cue) on the LVF would give rise to a 4 Hz pattern in attention performance. We did not find this pattern in our data: a 4-Hz peak was absent in the non-cued location on the LVF. A potential reason could be that attentional weight was still not equally divided across the two locations, which is likely as the probability division of a cue appearing was still 60% versus 40%. This unequal division could lead to more infrequent sampling of the non-cued location than the cued location, in turn resulting in a lack of a behavioural ‘footprint’ of rhythmic sampling at the non-cued location. An experiment involving a purely 50%–50% division (such as^5^), next to the currently used divisions, could shed light on this issue. However, that would remove the incentive to start sampling at the cued location, which was our aim when we decided to implement a 60%–40% division instead of a 50%–50% division. Given the inconsistencies of our findings with the main hypothesis as well as the difficulties in finding the main effect of our cue validity manipulation, we can at this moment only speculate about the absence of this effect.

An exploratory analysis found a non-significant 8 Hz peak for rhythmic attention at the non-cued locations after an invalid cue, at 80% cue validity. Originally, we expected an 8 Hz rhythmic sampling at both cued as well as non-cued locations (Fig. 1). At this moment, we can only speculate about these findings, especially considering that the analysis on the non-cued locations was exploratory. Previous studies have found conflicting effects of behavioural rhythms operating at attended locations^5,13^, but some studies only finding effects at non-attended locations^40,41^ (see also below). It is possible that overall attention is too strong at the attended location, rendering any rhythmic effect at the cued location not powerful enough to be detected, resulting in effects solely at the non-cued location. The 8 Hz effect at the non-cued location would reflect a periodic *interruption* of exploitative sampling at the cued location. Attention is then primarily deployed at the cued location, but periodic (8 Hz) windows provide an opportunity exploration towards the non-cued location. Alternatively, the phase reset due to the central cue could be less consistent when a cue is highly valid. This would result in more variable timing of the voluntary orienting leading to the absence of an effect for the 80% and 100% valid conditions. Again, these interpretations render new studies designed better at disentangling the effects of cued versus non-cued locations.

We found significant 4-Hz attentional rhythmicity in orienting towards the RVF, not towards the LVF. Instead, we found a non-significant ~ 7 Hz attentional rhythmicity towards the LVF. These differences between visual fields in these spectral patterns have been reported before^5,36^. As possible reason for the LVF-RVF dichotomy, Landau & Fries^5^ posed that an RVF flash more robustly causes a phase reset than an LVF flash. However, that is unlikely to explain the results in this study, as there was no spatial, hemifield-specific cue, but rather a centrally located cue. A phase reset from a centrally located stimulus would elicit a similar phase reset, regardless of target hemifield. Of course, the specific hemisphere by which we read out the rhythmic sampling behaviourally could be differentially sensitive to a phase reset or elicit a less consistent phase reset. Alternatively, the found rhythmic effect in the RVF, but not in the LVF, could be the result of an involvement of both hemispheres if the observed behavioural effects reflect widespread brain processes. Namely, there is left *and* right hemisphere involvement in attention towards the RVF, whereas attention towards the LVF largely only involves the right hemisphere^42–44^. Another alternative explanation is that the pathway from visual processing to responding is more direct (the Simon’s effect^45^) and that therefore task performance more directly reflects attentional sampling processes in the left hemisphere. This last hypothesis could be tested by repeating the experiment in a left-handed population.

Even though rhythmic attentional sampling has been found in a wide range of studies, most of these studies employed paradigms with exogenous cues. In such paradigms, visual attention is automatically drawn to the location of interest, by means of a spatial cue that appears on the same location as the upcoming target. A few studies showed rhythmic attentional effects after a centrally located cue, aimed at recruiting top-down, endogenous, orienting of attention^11,13,40,41^. Of these studies, only Helfrich et al.^13^ found rhythmic effects at the cued location, whereas Dugue et al.^11^ and Senoussi et al.^41^ only found rhythmicity at the *non-cued location* after an invalid cue (i.e. rhythmic *reorienting* of attention). Helfrich et al., next to their exogenous spatial cueing (Egly-Driver) task, employed a cued visual pop-out task with a centrally located arrow to show that rhythmic attention is independent of task structure^13^. In the task they found 4-Hz rhythmic attentional sampling (mapped onto frontoparietal neural synchronisation)^13^. We too implemented centrally located arrows, aimed to recruit endogenous attention and add to this scarce literature. However, studies have shown that arrows prompt attention shifts even when the cue probability is at chance level^46,47^. This indicates the presence of a learned and / or reflexive component involved in attentional orienting in response to an arrow cue. Alternatively, arrows elicit an independent type of orienting coined *automated symbolic orienting*. However, this reflexive cueing effect after an arrow is found to occur rapidly after the cue (i.e. below 300 ms) and that the observed endogenous effect stems from a concurrent processing of cue and target^48^. Note that the grating target in our study does not appear after at least 500 ms. It is thought that after 350 ms, the likelihood of cue-target conflict decreases, coinciding with a high probability that the cueing effect stems from volitional orienting^33^. Yet, we do not deny the controversy around the use of arrows for cueing. An experiment involving a symbolic cue associated with a visual field or location, such as an object in a certain colour or a word, could avoid the reflexive/voluntary orienting component that is thought to be recruited by arrow cues.

Another potential influence on our results could be the use of placeholders for our target stimuli. Placeholders have been found to affect inhibition of return (IOR), a phenomenon where target detection is first facilitated by the cue (around 0-300 ms) and then diminished (after 300 ms)^49^. For example, Taylor and colleagues found that the time course of the IOR phenomenon is altered when placeholders are used, with no IOR occurring at 200 ms with placeholders^50^. Studies which investigated longer cue-target intervals, found a generally larger IOR effect *with* than *without* placeholders (see^51^ for an overview). This suggests that the use of placeholders affects the rhythmic sampling behaviour as observed in our study. However, if IOR is contributed to by a rhythmic attentional sampling mechanism, as suggested before^4^, our study is better capable of capturing it than it would have without placeholders.

In recent years there has been criticism on the approach of using permutation tests for investigating behavioral rhythms in cognition as it leads to higher false positives when found effects are also influenced by aperiodic temporal structure or due (as hypothesized) to a periodic temporal structure^39^. Here, we try to minimize the effect of aperiodic noise by removing the linear trend from the data. We did analyse our data following the AR(1) analysis method proposed by Brookshire^39^, which fits the time series to an autoregressive model instead of permuting the trials in the time domain. We found a peak at 5.0 Hz, although it was not significant after multiple comparisons. Whereas a fair point is raised by Brookshire, there are important caveats to the analysis proposed. For example, the AR(1) method fails at detecting ‘true’ oscillatory patterns at lower frequencies (4–6 Hz) for a proportion of a simulated oscillation—notwithstanding behavioural oscillations contaminated with noise^52^. Furthermore, the scaling of the AR(1) surrogate date leads to an inflation of its power and therefore increases the false positive rate^53^. Therefore, given the shortcomings related to AR(1) analysis, the fact that we could not replicate our results with this method does not directly invalidate our results.

Our core finding is a 4 Hz rhythmic sampling of cued locations in the RVF that only occurs when cue validity is low (60%), but not when cue validity is high (80%). Unfortunately, we could not unmistakably show that our task manipulations altered overall attentional resource allocation. That is, we did not find a significant effect of cue validity in the RVF, but only in the LVF, and we found that attentional reorienting cost (across visual fields) is significantly lower at lower cue validity (60%) than high cue validity (80%). As it stands, our results show that rhythmic sampling does depend on task conditions and/or instructions. Importantly, our finding of a 4 Hz effect in the RVF is consistent with previous findings that show 4 Hz sampling only in the RVF^5,36^. Additionally, our study is one of the first to show rhythmic sampling effects using centralized symbolic, rather than strong lateralized cues.

### Supplementary Information

Supplementary Information.

## Data Availability

The analysis code and data supporting the findings in this article are available at https://github.com/olofvanderwerf/rhythmic-attention. The Jupyter Notebooks containing the analysis can be directly opened, explored, and executed interactively through Binder (see “readme” on GitHub for direct links to interactive Jupyter Notebooks).
