# Supplementary material for: Investigating the role of task relevance during rhythmic sampling of spatial locations
Source: Sci Rep. 2023 Aug 5;13:12707. doi: 10.1038/s41598-023-38968-z (PMC10404272; doi:10.1038/s41598-023-38968-z)
Supplement: Supplementary file 1 — Supplementary Information. [file 41598_2023_38968_MOESM1_ESM.docx]

SUPPLEMENTARY METHODS

Next to the single-trial least squares spectrum (stLSS) analysis, we performed a Fast Fourier Transform (FFT) and analysed the power spectrum using permutation testing. Below, we describe the pre-processing, analysis, and statistical steps for this FFT method.

*Pre-processing.* Trials with blinks (no data for more than 100 ms) and saccades (exceeding 2° of visual angle), and trials with incorrect responses were excluded from the analysis (84 trials (7%**)** on average). In every time bin between ~516.67 and 1650 ms, in steps of ~16.67 ms, all reaction times of that time bin were averaged together with two adjacent time bins. The resulting time courses were detrended by fitting a second order polynomial. We created a time course of reaction times over cue-target intervals for cue type (valid vs. invalid) and visual field (LVF, RVF), for each participant separately (**Supplementary figure 3a-b)**.

*Analysis.* We group-averaged the time courses for each cue validity condition and visual field separately. Time courses were multiplied by a Hamming window and zero-padded to reach a length of 10 seconds. For each condition, we applied a Fast Fourier Transform over these pre-processed time series, resulting into a single amplitude spectrum.

*Statistics.* To investigate statistical significance of the found spectral peaks, we applied non-parametric permutation testing. First, we tested whether, for each cue validity condition, there was a significant temporal pattern in the time courses. Here, the null hypothesis states that there is no temporal pattern, thus we shuffled the temporal bin labels. Second, we tested whether rhythmic attention is different across cue validity conditions. Here, the null hypothesis states that there is no difference in cue validity, thus we shuffled the condition labels of the valid trials, for each visual field separately. We repeated this 1000 times, so that for each participant and each visual field there was a surrogate distribution of shuffled temporal bin labels and condition labels. We applied the same analysis steps (see above) and ended up with a surrogate distribution of 1000 power spectra, to which we could compare the observed power spectrum. For each frequency between 2 and 20 Hz, we determined the proportion of values in the surrogate distribution that exceeded the power spectrum (i.e. the p-value) and corrected the resulting p-values for multiple comparisons using the False Discovery Rate (Benjamini & Hochberg).

SUPPLEMENTARY RESULTS

### The spectral profile of RVF attentional performance over time revealed a significant spectral peak at ~4 Hz (range: 3.9 – 4.7 Hz, *P*_peak_ = 0.03, FDR-corrected, at 4.2 Hz) for the 60% cue validity condition, but no significant peaks for the 80% or 100% cue validity conditions (all *P* > 0.10, figure 2D). Periodicities in attentional performance on valid trials in the LVF showed a peak at 7.1 Hz in the 60% cue validity condition, albeit not significant after FDR-correction (*P*_peak_ = 0.09, FDR-corrected, Supplementary Figure 3c). As with the RVF, no significant peaks were found for the 80% and 100% cue validity conditions in the LVF (all *P* > 0.10, Supplementary Figure 3d).

We tested more directly whether rhythmic attention was dependent on cue validity. To this aim, we employed non-parametric permutation testing where we shuffled the cue validity condition labels (60%, 80%, 100%) across trials. In the RVF, we found a significant peak at ~4.5 Hz in the spectral profile in the 60% cue validity condition compared to the surrogate distribution (4.4 – 4.8 Hz, *P*_peak_ < 0.001), further corroborating the specificity of our findings to low validity at RVF only. We did not find any statistically significant peaks in the other cue validity conditions in the RVF (all *P* > 0.10), nor any peaks in the LVF (all *P* > 0.10).

SUPPLEMENTARY FIGURES


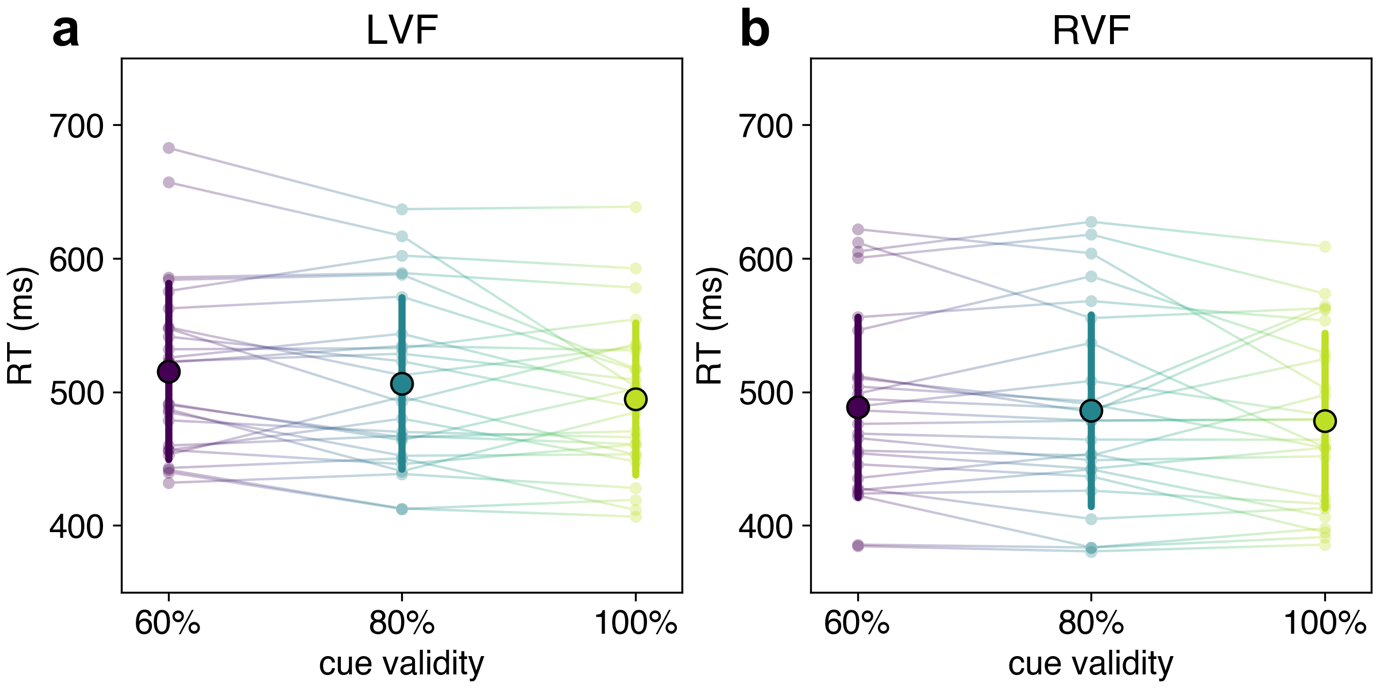


**Supplementary figure 1: Reaction times per condition, per visual field.** (a-b) Reaction times on valid trials for each cue validity condition, separated for the left visual field (LVF, in a) and right visual field (RVF, in b). For the LVF, post-hoc analysis revealed a significant difference between the 60% and 100% cue validity conditions (*P* = 0.005).

**
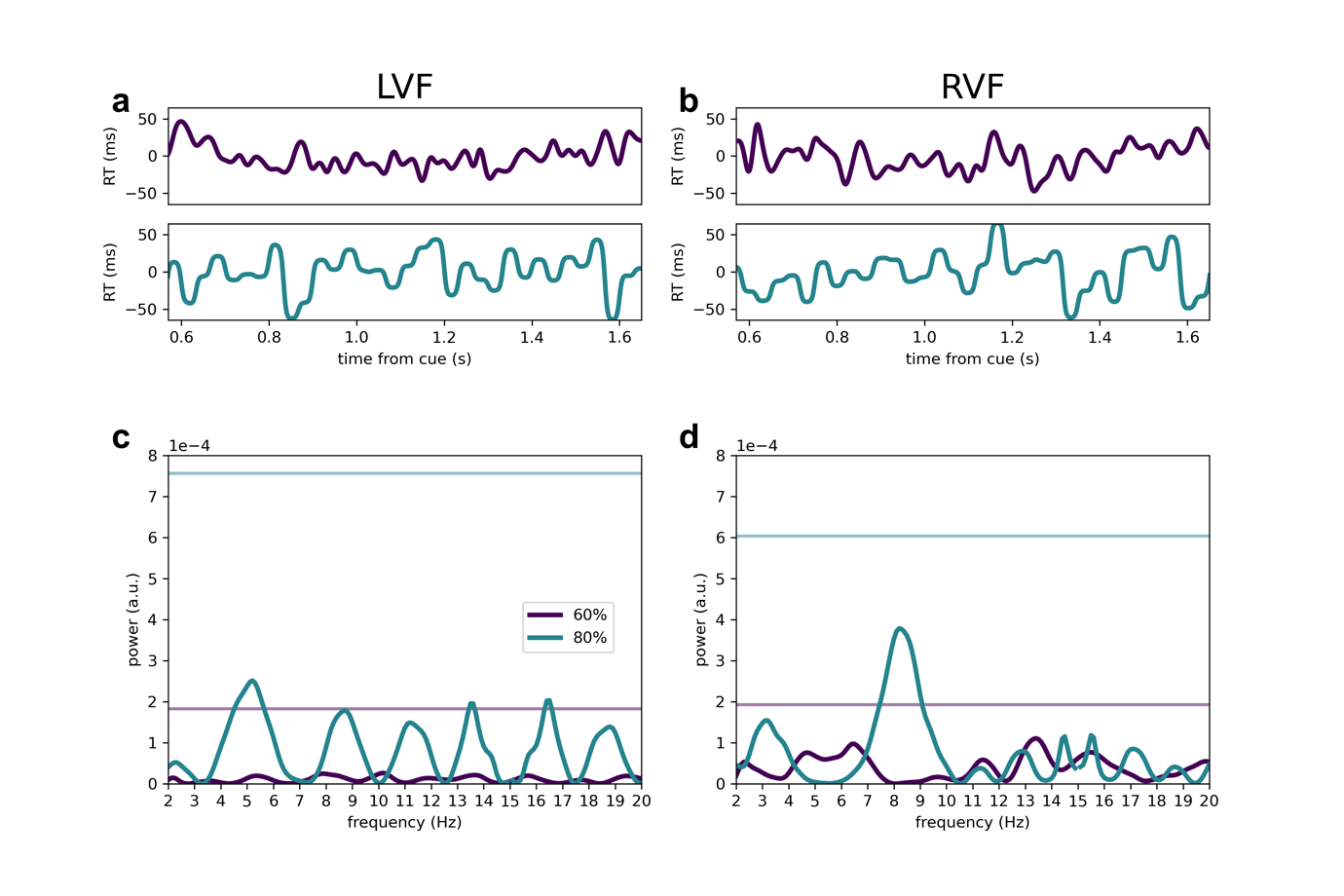
**

**Supplementary figure 2: Rhythmic sampling at invalid locations.** (a-b) Time courses of reaction times across cue-to-target intervals (mean ± standard error of measurement (SEM)) at invalidly cued locations on the LVF (a) and the RVF (b). Time courses are constructed using a Gaussian moving kernel across cue-to-target intervals. (c-d) Power spectra respectively corresponding to the time courses in a and b, as yielded from the stLSS analysis. Horizontal lines in corresponding colours denote the 95^th^ percentile of the maximum permutation spectrum (i.e. Max-Based correction for multiple comparisons). The non-diffuse manner at which invalid LVF and RVF trials were distributed across time bins at 80% cue validity introduced a sampling artifact at 15.0 Hz, which we left out in this visualisation. This sampling artifact does not occur in the 60% cue validity condition, nor in the pooled data at invalid trials (**figure 3**). In these conditions, trials were uniformly distributed across time bins.


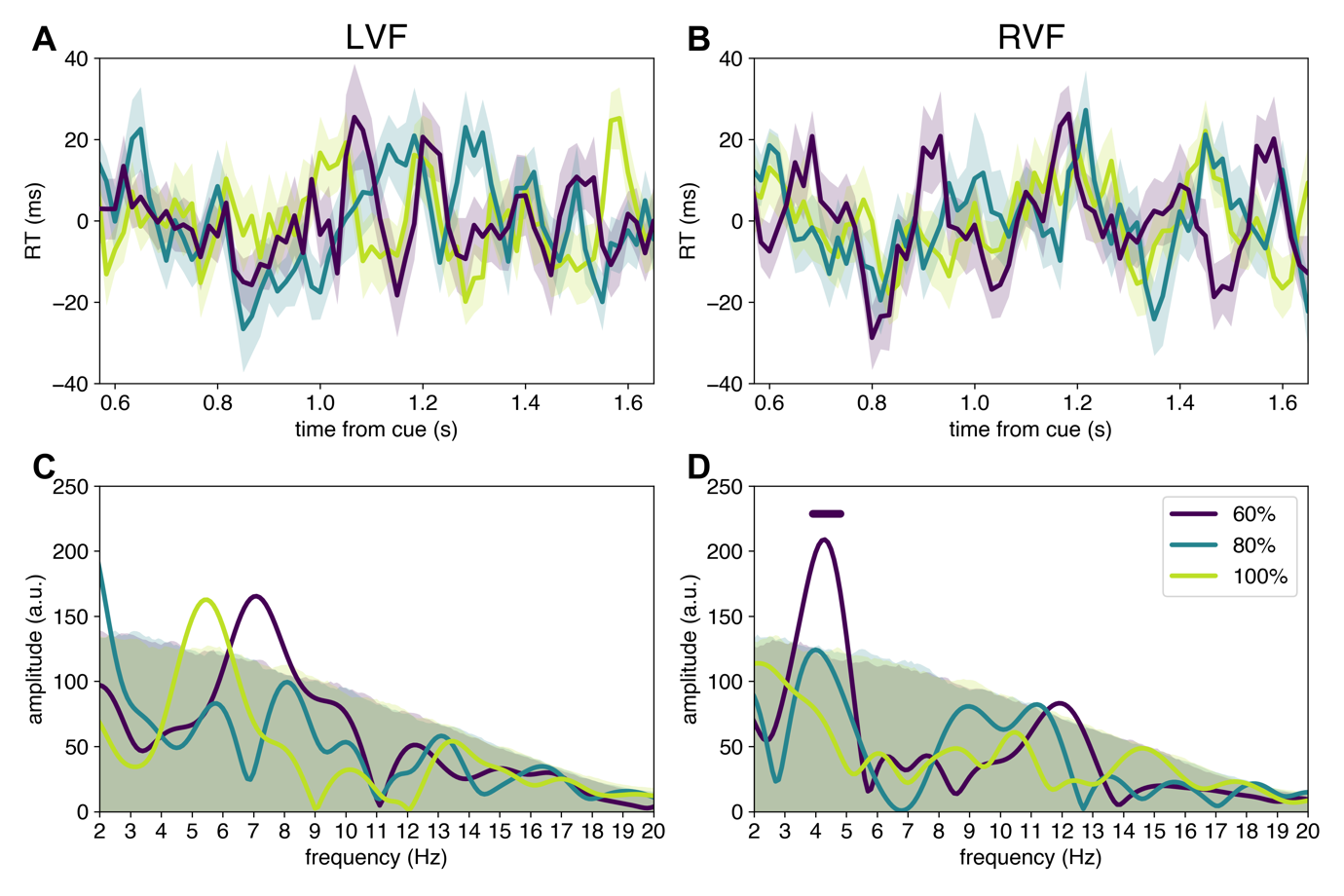


**Supplementary figure 3** (a-b) Constructed time courses of reaction times across cue-to-target intervals (mean ± standard error of measurement (SEM)) at validly cued locations on the LVF (a) and the RVF (b). (c-d) Power spectra respectively corresponding to the time courses in a and b. Shaded area denotes the 95^th^ percentile of the surrogate distributions (not FDR-corrected). There is a significant ~4 Hz peak in the 60% condition on the RVF (*P*_peak_ = 0.03 at 4.2 Hz).
